# Professional Pressure

**Published:** 1889-12-14

**Authors:** 


### Editor's Letter-Box.

Our Correspondents are reminded that prolixity is a great bar to publication, and that brevity of style and conciseness of statement greatly facilitate early insertion.]

### PROFESSIONAL PRESSURE.

You deserve the sincere thanks of the medical profession for your excellent article "The Doctor's Pulpit," and I hope the paper will produce an effect equivalent to its worth. I think that every doctor, even of moderate ability, can act without detriment to himself or to his patients on the principles you have laid down.

What is the daily routine of nearly every medical man in large practice? It is a constant rush from 9 a.m. to 9 or 10 p.m., with irregular meals, and little comfort, and after this, perhaps, an outing through the night. The effect of this is the rotting and wearing out at a rapid rate of the medical man's bodily and mental faculties, and we thus account for his average shortness of life. But the doctor never thinks that this mode of action is also a blind form of suicide. Man is an animal who must be amused, as well as worked, but a great mass of doctors have little time for recreation or reading. Except that his is a life of self-sacrifice and constant adherence to duty (?) what other respect can such an isolated individual expect from his fellows? A man of one book, and I believe it is from the tendency to "grasp" that this principle of laying oneself out to constant work is carried on. But, happily, to those keen enough to see it, and bold enough to make a stand for legitimate independence, and for the honour and exaltation of their profession, the remedy is at hand, and if they accept it, they are certain to work considerably longer, and with as much comfort and satisfaction as is possible in the exercise of what is always a laborious calling. In order, if possible, to induce others of my professional brethren to try what I have done, I beg to state my case.

I am an average practitioner, with a large practice, and I am glad to say on good terms with all my respectable professional neighbours. I do all my consultations before 11 a.m., but, if I had clubs, I would also consult between 6 and 7 p.m., I attend only to urgent emergencies after 6 p.m. Most of my patients let me know before 11 a.m. if they want to be visited, because I rarely alter my list after it is made out. This rule cannot be carried out without time and careful managing, but I have found that if it is explained to people that it has been found that work cannot be done on other principles without too great hurry and serious injury to health, they will see the reason of the position at once. Who else but a doctor attempts such things? I am glad to state that the result of what I have done has been no loss to me pecuniarily, but great gain, so far as comfort and the capacity for profitable work is concerned. It is well known that people seek with great eagerness services difficult to get, value them much more, and, I am sure, pay the bills better of the men whom they consider more or less independent of them. I would commend this last statement to greedy men for their most earnest consideration. Daylight, also, is most important, so far as light and colour is concerned, for seeing people, and patients supposed to be ailing, coming out at night, especially in winter, tends to neutralise the effects of even good medicine. Your plan of consulting between 6 p.m. and 7 p.m. would cover the whole ground. I believe I lose some City men, but not many. They see me before they go to business, if they put much value on my services. If they don't, then they are better with somebody else. The combination you speak of would settle the matter at once, but I am of your opinion, that this happy time is rather distant. However, I have proved to my entire satisfaction that the principle you so well state can be carried out by individuals.

In the country, where there is no opposition, the rule is carried out by many friends of mine in good practice, and there are no men more respected in life, or more missed at death, than good class country doctors. There is no use blinding oneself to the fact that there are amongst our ranks many "rankers," but there are, also, in every district some respectable and honourable practitioners; and if these would strongly combine they could, to a very great extent, command the good behaviour of those who do not respect the traditions of their profession. I doubt if there is any other class of men so loyal to one another, or so faithful and generous to those they deal with, as good class doctors, who have had certain traditions instilled into them by their teachers, and by their living in the atmosphere of the high-class schools through which they get their degrees or qualifications. They must begin practice with their principles fixed; and I think, with you, that those high in the ranks of the profession should consider it their duty to confer the greatest boon possible on their students by constantly bringing before them the ethics of medicine, and by telling them of the futility of pandering to public tastes, or working on any unstable or shady principles which are, in the long run, inevitably fatal.

Spectator.

